# Arsenic Impairs Differentiation of Human Induced Pluripotent Stem Cells into Cholinergic Motor Neurons

**DOI:** 10.3390/toxics11080644

**Published:** 2023-07-25

**Authors:** M. Chiara Perego, Benjamin D. McMichael, Nicholas R. McMurry, Scott W. Ventrello, Lisa J. Bain

**Affiliations:** 1 Department of Biological Sciences, Clemson University, Clemson, SC 29634, USA; 2 Department of Biology, University of North Carolina, Chapel Hill, NC 27599, USA

**Keywords:** arsenic, human induced pluripotent stem cells, motor neurons, ChAT, cholinergic synapse

## Abstract

Arsenic exposure during embryogenesis can lead to improper neurodevelopment and changes in locomotor activity. Additionally, in vitro studies have shown that arsenic inhibits the differentiation of sensory neurons and skeletal muscle. In the current study, human-induced pluripotent stem (iPS) cells were differentiated into motor neurons over 28 days, while being exposed to up to 0.5 μM arsenic. On day 6, neuroepithelial progenitor cells (NEPs) exposed to arsenic had reduced transcript levels of the neural progenitor/stem cell marker nestin (*NES*) and neuroepithelial progenitor marker *SOX1*, while levels of these transcripts were increased in motor neuron progenitors (MNPs) at day 12. In day 18 early motor neurons (MNs), choline acetyltransferase (CHAT) expression was reduced two-fold in cells exposed to 0.5 μM arsenic. RNA sequencing demonstrated that the cholinergic synapse pathway was impaired following exposure to 0.5 μM arsenic, and that transcript levels of genes involved in acetylcholine synthesis (CHAT), transport (solute carriers, *SLC18A3* and *SLC5A7*) and degradation (acetylcholinesterase, *ACHE*) were all downregulated in day 18 early MNs. In day 28 mature motor neurons, arsenic significantly downregulated protein expression of microtubule-associated protein 2 (MAP2) and ChAT by 2.8- and 2.1-fold, respectively, concomitantly with a reduction in neurite length. These results show that exposure to environmentally relevant arsenic concentrations dysregulates the differentiation of human iPS cells into motor neurons and impairs the cholinergic synapse pathway, suggesting that exposure impairs cholinergic function in motor neurons.

## 1. Introduction

Arsenic is a well-known environmental contaminant and one of the most abundant trace elements found in the Earth’s crust [1,2]. Arsenic has been classified as one of the most hazardous chemicals in the world [1] as it poses a threat to 150 million people that are exposed to arsenic through drinking contaminated groundwater [2,3]. The current drinking water standard established by the World Health Organization and US Environmental Protection Agency is 10 ppb. However, arsenic concentrations much higher than the drinking water standard have been reported in China, Mexico, Chile, Argentina, India, Bangladesh, and the U.S. [1,4,5,6,7].

Several human epidemiological studies have shown that arsenic exposure during embryonic development and the perinatal period impairs neurodevelopment, leads to behavioral alterations, and is associated with impaired cognitive function, decreased IQ, and disrupted memory and learning skills [8,9,10,11,12]. Similarly, impaired developmental and behavioral alterations have also been observed in rodent pups when dams were exposed to inorganic arsenic during pregnancy [13,14,15]. For instance, exposure to 50 ppb arsenic during embryonic development led to impaired hippocampal neurogenesis and the downregulation of genes involved in neurite elongation [16]. In vitro, arsenic inhibits neuronal differentiation and reduces neurite outgrowth in P19 stem cells, PC12 cells, Neuro-2a cells, differentiated SH-SY5Y cells, and in primary neurons [17,18,19,20,21,22].

Functionally, arsenic exposure alters dopaminergic, glutaminergic, and cholinergic neurotransmitter pathways in the brain (reviewed in [23,24]). For example, arsenic can reduce acetylcholine (ACh) levels and acetylcholinesterase (AChE) activity in the cerebral cortex [25,26,27,28] while arsenic exposure to pregnant rats resulted in reduced choline acetyltransferase (ChAT) protein levels and reduced AChE activity in the brains of the pups [29,30].

While much is known about arsenic’s effects on neurons in the brain, relatively few studies have examined its effects in the peripheral nervous system, particularly in motor neurons. A recent epidemiological study analyzed the link between motor neuron disease and heavy metals released into rivers showing a 16.7% higher risk of mortality associated with motor neuron disease in arsenic-contaminated areas [31]. Symptoms often listed in epidemiological studies of arsenic-exposed populations include muscle weakness, muscle atrophy, or reduced motor function [32,33,34,35]. Further, changes in arsenic methylation capacity are associated with changes in muscle function [36]. In rodents, arsenic-exposed pups and adults have reductions in locomotor activity and grip strength [28,30,37]. Zebrafish exposed to extremely high arsenic concentrations (200 ppm) have altered numbers of motor neurons [38].

The purpose of the current study was to determine whether an environmentally relevant arsenic exposure to human induced pluripotent stem (iPS) cells impaired their differentiation and/or functioning into motor (cholinergic) neurons. Our results showed that exposure to arsenic during motor neuron differentiation alters mRNA expression of neural progenitor markers (*SOX1* and *NES*), and motor neuron progenitor markers (*OLIG).* Further, significant downregulation of genes involved in the acetylcholine cycle (CHAT, *SLC18A3*, *SLC5A7*, *ACHE*) was all seen in early motor neurons that were exposed to arsenic. Finally, protein expression of neural marker MAP2 and motor neuron marker ChAT was decreased concomitantly with a reduction in neurite length in late motor neurons exposed to arsenic. These results suggest that arsenic exposure delays the differentiation of human iPS cells into motor neurons and dysregulates the acetylcholine cycle.

## 2. Materials and Methods

### 2.1. HiPS Cell Culture and Arsenic Exposure

Human iPS cells (DYS0100, ATCC, Manassas, VA, USA) were cultured in mTeSR1 medium (StemCell, Vancouver, BC, Canada) on 6-well plates coated with Matrigel (Corning, Corning, NY, USA). Cells were maintained in a humidified incubator at 37 °C and 5% CO_2_ and medium was changed daily.

To determine appropriate exposure concentrations, on Day -1, human iPS cells were dissociated with ReLeSR (StemCell) and plated on 6-well Matrigel-coated plates (2 × 10^5^ cells/well) with mTeSR1 medium containing 10 µM Y-27632 ROCK inhibitor (Tocris, Bristol, UK). From Day 0 to Day 6, cells were cultured in mTeSR1 medium containing 0, 0.1, 0.25, or 0.5 µM arsenic as sodium arsenite (Sigma Aldrich, St. Louis, MO, USA), with daily medium changes. At day 6 (D6), cells were collected for flow cytometry or stored at −80 °C in TRIzol (Sigma Aldrich) for subsequent RNA extraction. Control and arsenic-exposed samples were cultured as independent replicates (*n* = 3–6 for each treatment).

### 2.2. Cell Differentiation

To investigate arsenic effects on motor neuron differentiation, human iPS cells were induced to differentiate into motor neurons as previously described [39,40]. Briefly, cells were dissociated with Dispase (1 mg/mL) (Millipore Sigma, St. Louis, MO, USA) and split 1:6 onto Matrigel-coated 6-well plates (Day -1). Cells were allowed to attach for 24 h and cultured in mTeSR1 medium supplemented with 10 µM Y-27632 ROCK inhibitor. The medium was switched, and cells were cultured in neural medium composed of DMEM/F12 (Gibco, Waltham, MA, USA) and Neurobasal medium (Gibco) at 1:1, 0.5xB27 (Gibco), 0.5x N2 (Gibco), 0.1 mM ascorbic acid (Fisher, Waltham, MA, USA), 1x glutamine (Gibco) and 1x Pen/Strep (Corning) for 28 days supplemented with different growth factors (Supplementary Table S1). To induce neuroepithelial progenitors (NEPs; Day 6), cells were cultured in the neural medium supplemented with 3 μM CHIR99021 (StemCell), 2 μM DMH-1 (Tocris), and 2 μM SB431542 (Sigma Aldrich) for 6 days performing a media change every other day. To induce motor neuron progenitors (MNPs; Day 12), NEPs were dissociated with Dispase (1 mg/mL) and split 1:6 onto Matrigel-coated 6-well plates. Cells were cultured in the neural medium supplemented by 0.1 μM retinoic acid (Acros Organics, Geel, Belgium), 0.5 μM purmorphamine (Millipore Sigma), 1 μM CHIR99021, 2 μM DMH-1 and 2 μM SB431542 for an additional 6 days, performing a media change every other day. Subsequently, to induce early motor neurons (early MNs; Day 18), MNPs were dissociated with Dispase (1 mg/mL), split 1:6 onto Matrigel-coated 6-well plates, and cultured in neural medium supplemented with 0.5 μM retinoic acid and 0.1 μM purmorphamine for 6 days performing a media change every other day. Finally, to induce mature motor neurons (mature MNs; Day 28), early MNs were dissociated with Accumax (eBioscience, San Diego, CA, USA), plated on Matrigel-coated 6-well plates and cultured for 10 days in neural medium supplemented by 0.5 μM retinoic acid, 0.1 μM purmorphamine, 0.1 μM Compound E (Millipore Sigma), 10 ng/mL insulin-like growth factor 1 (IGF-1) (Sigma Aldrich), 10 ng/mL brain-derived neurotrophic factor (BDNF) (R&D Systems, Minneapolis, MN, USA) and 10 ng/mL ciliary neurotrophic factor (CNTF) (R&D Systems). Throughout the differentiation process, cells were exposed to 0, 0.25, and 0.5 µM arsenic as sodium arsenite. Representative cell images were captured on VistaVision microscope (VWR) with 10X or 40X objectives. NEPs, Olig2+ MNPs, and early MNs were harvested and stored at −80 °C in TRIzol for subsequent transcript analysis and RNA sequencing, while early MN and mature MNs were fixed in 4% paraformaldehyde for subsequent immunohistochemical analysis. Control and arsenic-exposed samples were cultured as independent replicates (*n* = 3–6 for each treatment).

### 2.3. In Vivo Arsenic Exposure and Hippocampi Collection

Adult C57/BL6 mice (Taconic) were exposed to 0 or 100 ppb of arsenic as sodium arsenite in their drinking water for five weeks as previously described ([41]; *n* = 6 per exposure group). Approval to conduct this study was obtained from Clemson’s Institutional Animal Care and Use Committee (IACUC). No behavior, motor, or weight changes were noted in the mice. Following five weeks of exposure, mice were euthanized, the hippocampi harvested, fixed in formalin, and embedded in paraffin for subsequent immunohistochemical analysis.

### 2.4. Quantitative PCR

RNA was extracted using TRIzol and a Nanodrop (Thermo Fisher) was used to determine RNA concentration and purity. M-MLV reverse transcriptase (Promega, Madison, WI, USA) was used to convert extracted RNA (2 µg) into cDNA. Gene expression was assessed by real-time quantitative PCR on a Bio-Rad iQ5 thermocycler (Hercules, CA, USA) using RT^2^ SYBR Green (Applied Biosystems, Foster City, CA, USA) and 10 μM forward and reverse gene-specific primers (Supplementary Table S2). Samples were run in triplicate and qPCR run efficiency was determined using a standard curve generated with five concentration points (10^−3^–10^−7^ ng cDNA). Absence of non-specific primer binding was verified through a melt curve obtained for each analysis. Data were normalized to housekeeping genes Gapdh and β2-microglobulin. Gene expression was analyzed using the delta-delta Ct method, setting the mean of the control group set to 1 and calculating fold changes in the treatment groups [42].

### 2.5. Immunohistochemistry

D18 early MNs and D28 mature MNs were exposed to 0.5 μM arsenic and cultured on Matrigel-coated glass-bottom culture Wilco dishes as described above. Cells were fixed in 4% paraformaldehyde, permeabilized with 0.2% Triton X-100 in PBS, and blocked in PBST with 5% BSA and 10% donkey serum for one hour at room temperature. Primary antibodies for ChAT (1:100, Millipore Sigma, #AB144P) and MAP2 (1:200, Millipore Sigma, #AB5622) were incubated overnight at 4 °C. After incubation with Alexa Flour 488 secondary antibodies (1:500, anti-goat, Invitrogen, Waltham, MA, USA, #A11055) and Alexa Fluor 594 (1:500 anti-rabbit, Invitrogen, #A21207), nuclei were counterstained with DAPI (1:1000, Invitrogen, #D1306). Samples were imaged using a Leica DMi8 widefield microscope system (Leica Microsystems, Buffalo Grove, IL, USA), equipped with a Leica DFC9000 GTC camera and a 20X dry objective (N.A. = 0.4). To image DAPI, we used a LED_405 filter cube with excitation wavelength of 405/60 and emission wavelength of 470/40 nm. To image Alexa Fluor 488, we used a GFP-T filter cube with excitation wavelength of 475/40 nm and emission wavelength of 530/50 nm. To image Alexa Fluor 594, we used a Cherry-T filter cube with excitation wavelength of 560/40 and emission wavelength of 630/75 nm. Camera exposure times and gain settings for imaging Alexa Fluor 488 and Alexa Fluor 594 were kept consistent within each experiment time point, although there is some variability between experiment time points. Similarly, all images within each time point were exported as. TIF uses the same lookup table settings. The system software was Leica LAS-X, Version 3.6.0.20104 (Leica Microsystems). Images were analyzed in ImageJ (v 14) to determine protein expression. For D18 early MNs, ChAT, and MAP2 expression were determined by assessing the integrated density value (IDV), which was normalized to area. For D28 mature MNs, cell bodies were selected to determine IDVs. Protein expression was assessed for two to six cell bodies per biological replicate.

Hippocampi harvested from adult mice were fixed overnight in NBF, dehydrated in ethanol, embedded in paraffin, and sectioned at 10 μm. Slides were deparaffinized and Tris-EDTA buffer (pH 9) was used for antigen retrieval. Primary antibodies for ChAT (1:200, Millipore Sigma, #AB144P) and MAP2 (1:500, Millipore Sigma, #AB5622) were incubated overnight at 4 °C. After incubation with Alexa Flour 488 secondary antibodies (1:500, anti-goat, Invitrogen, #A11055) and Alexa Fluor 594 (1:500 anti-rabbit, Invitrogen, #A21207), nuclei were counterstained with TO-PRO-3 (1:1000, Thermo Fisher, #T3605). Samples were imaged using a Leica DM2500 confocal microscope (Leica Microsystems, Buffalo Grove, IL) using a 20X objective (N.A. = 1.4) and a zoom of 1.0. To image TO-PRO-3, we used an excitation wavelength of 635 nm and collected emission wavelengths of 641 to 699 nm, with a time gate of 0.5 to 6.0 ns, and a frame average of 2. To image Alexa Fluor 488, we used an excitation wavelength of 488 nm and collected emission wavelengths of 504 to 568 nm, with a time gate of 0.5 to 6.0 ns and a frame average of 4. To image Alexa Fluor 594, we used an excitation wavelength of 532 nm and collected emission wavelengths of 590 to 633 nm, with a time gate of 0.5 to 6.0 ns and a frame average of 4. Images were analyzed in ImageJ to determine protein expression and distribution. Integrated density value (IDV), normalized to the area, was used to determine the expression of ChAT and MAP2.

### 2.6. Sholl Analysis

To assess neurite length and dendritic patterns, Fiji (ImageJ) was used. Neural processes of MAP2+ D28 MNs were traced, and their length was measured using Simple Neurite Tracer (SNT) plug-in. Sholl analysis was performed to investigate neuronal branching and assess the number of dendritic intersections at 1μm intervals, using the Sholl plug-in.

### 2.7. RNA Sequencing

RNA sequencing was performed by Novogene (Sacramento, CA, USA) on rRNA-depleted RNA extracted from day 6 NEPs and day 18 early MNs (*n* = 3 for each treatment). Paired-end sequencing was performed via the Illumina platform and reads of 150 bp length were checked for quality metrics and trimmed using BBDuk (v38.96) for low-quality bases using a quality score of 20 [43]. Paired-end clean reads were aligned to the *Homo sapiens* reference genome GRCh38 using STAR aligner and BBTools (v2.7.7a) [44]. Reads mapping to genes were counted using featureCounts (v.2.0.3) [45]. Differential expression analysis was conducted using the DESeq2 (v1.29.7) R package [46] using the Wald test to compare control and arsenic-exposed cells at D6 and D18. The Benjamini–Hochberg adjustment was used to control for false discovery rate (FDR). Functional annotation analysis was performed using the R package annotables [47] on differentially expressed genes with an adjusted *p* < 0.05. The hierarchical clustering distance method was performed to cluster samples using the pheatmap package in R [48]. The R package clusterProfiler was used to run Gene Ontology (GO) gene set enrichment analysis (GSEA) of differentially expressed genes [49,50]. GO terms with adjusted *p* < 0.05 were considered significantly enriched by differentially expressed genes. Lastly, biochemical pathways disrupted following arsenic exposure during motor neuron differentiation were identified using the clusterProfiler package to assess the statistical enrichment and over-representation of differential gene expression in KEGG pathways.

### 2.8. Flow Cytometry

Annexin V and SyTox green staining was performed to determine cell viability and apoptosis in human iPS cells and D6 NEPs following arsenic exposure. Briefly, cells were incubated in Accumax at 37 °C for 10 min to create a single cell suspension, and resuspended in FACS buffer (1X PBS, 1% BSA, 0.1% sodium azide). Cells were incubated with Annexin V (1:200, Santa Cruz, Santa Cruz, CA, USA #sc-74438) at 4 °C for 30 min followed by incubation with Alexa Fluor 568 secondary antibody (1:400, anti-mouse, Invitrogen, #A11004) at 4 °C for 30 min. Subsequently, cells were washed in FACS buffer and incubated with SyTox Green (1:1000, Invitrogen, #S34860) for 20 min. Flow cytometry was performed using a Bio-Rad S3e Cell Sorter. SyTox green fluorescence was assessed at 488 nm excitation and 504 nm emission while Annexin V fluorescence was assessed at 561 nm excitation and 603 nm emission.

### 2.9. Statistical Analysis

Results are expressed as mean ± SE using Graphpad Prism 9 (San Diego, CA, USA). Statistical significance for differential gene expression was calculated by Student’s *t*-test or by ANOVA followed by Tukey’s multiple comparison test. Principle coordinate analysis (PCA) was conducted with the transcript data for each individual well in GraphPad Prism 9. Protein expression for immunohistochemistry was determined using ImageJ. Statistical significance for average protein expression in control and arsenic-treated biological replicates was assessed by Student’s *t*-test. Neurite length was determined using neurite tracer plug-in to examine MAP2^+^ neural processes of one to two cells per biological replicate. Sholl analysis was conducted at 1 μm intervals, using the Sholl plug-in for one to two cells per biological replicate. Statistical significance for average neurite length and number of dendritic intersections in control and arsenic-treated samples was assessed by Student’s *t*-test. Percentage of live, apoptotic, and necrotic cells was investigated by flow cytometry. Statistical significance for average percentage of live, apoptotic, and necrotic cells in control and arsenic-treated samples was assessed by two-way ANOVA followed by Tukey’s multiple comparison test. Data were analyzed for normal distribution and *p* < 0.05 was considered statistically significant.

## 3. Results

### 3.1. Human iPS Cells Are Sensitive to Arsenic Exposure

**Figure 1:**
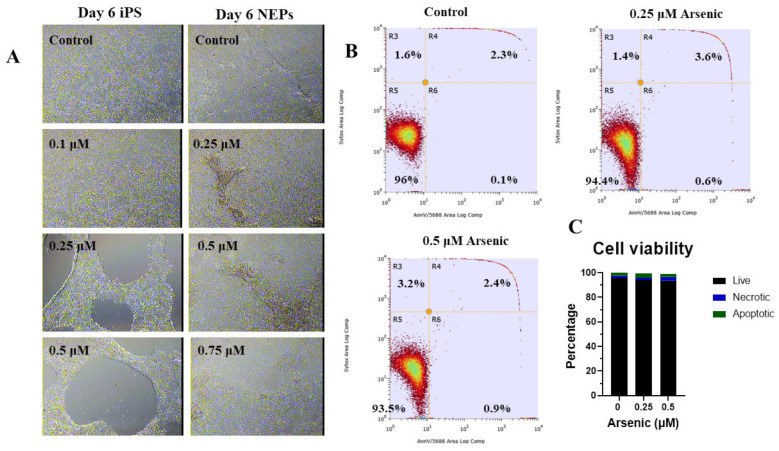
Low arsenic levels induce cell death in human iPS cells, but not in neuroepithelial progenitor cells (NEPs). Representative images of human iPS and NEP cells exposed to 0 arsenic for six days taken at 10X (**A**). Representative flow cytometry analysis of day 6 NEPs cells exposed to 0, 0.25, or 0.5 μM arsenic. Cells were stained with Annexin V and SyTox green to assess apoptosis. Text shows the percentage of live (R5), early apoptotic (R6), late apoptotic (R4), and necrotic (R3) cells reported in each quadrant (**B**) Percentage of live, apoptotic, and necrotic NEPs cells exposed to 0, 0.25 or 0.5 μM arsenic (*n* = 3 per exposure group) (**C**). Statistical differences were determined using two-way ANOVA followed by Tukey’s multiple comparison test (*; *p* ≤ 0.05).

To determine appropriate arsenic concentrations to use during differentiation, human iPS cells were first cultured for six days in an mTeSR medium to maintain them as stem cells in their pluripotent state. During this maintenance phase, cells were treated with 0, 0.1, 0.25, and 0.5 µM arsenic (equivalent to 7.5–37.5 ppb). Morphological analysis indicates that 0.25 and 0.5 µM concentrations result in significant cell death starting at day 4 and continuing to day 6 (Figure 1A). qPCR analysis of the cells exposed to 0.5 µM As showed increased expression of pluripotency transcripts, including *SOX2* (Figure S1A), *POU5F1* (Figure S1B), and *NANOG* (Figure S1C). Consistently, we observed a strong positive correlation of *SOX2*, *POU5F1,* and *NANOG* transcript levels in human iPS cells (Supplementary Table S3).

Next, iPS cells were differentiated into neuroepithelial progenitors (NEPs) for six days while being exposed to 0, 0.25, 0.5, or 0.75 µM arsenic. There were no morphological differences between exposure groups (Figure 1A). Consistently, flow cytometry with Annexin V and Sytox green (Figure 1B) did not indicate significant differences in the percentage of live, apoptotic, and necrotic cells following exposure to 0.25 or 0.5 μM arsenic for six days (Figure 1C). Considering the absence of morphological alterations during NEP differentiation and no significant changes in cell viability or death, concentrations of up to 0.5 μM arsenic were used for the remainder of the study.

### 3.2. Differential Gene Expression of Key Pluripotency and Differentiation Markers Confirms Generation of Motor Neurons (MNs)

**Figure 2:**
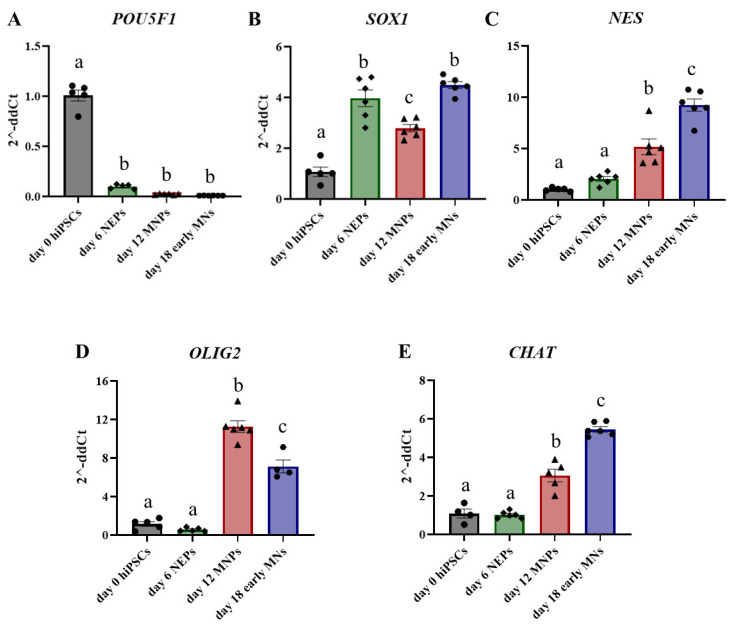
Differential gene expression of key markers during differentiation of human iPS cells into motor neurons. Transcript levels of *POU5F1* (**A**), *SOX1* (**B**), *NES* (**C**), *OLIG2* (**D**), and CHAT (**E**) were assessed by qPCR in control samples from day 0 human iPS cells (gray, circles), day 6 NEPs (green, diamonds), day 12 MNPs (red, triangles) and day 18 early MNs (blue, circles). Fold change was determined using the ΔΔCt method, and results were normalized to geometric mean of Gapdh and β2-microglobulin. Statistical differences were determined using ANOVA followed by Tukey’s multiple comparison test (*p* ≤ 0.05; *n* = 4–6 per exposure group; a = no statistical difference from day 0; b = statistically different from day 0; c = statistically different from all other days).

To confirm the differentiation of human iPS cells into day 6 (D6) NEPs, day 12 motor neuron progenitors (MNPs), and day 18 early motor neurons (MNs), we analyzed gene expression of key stage-specific markers in control samples throughout the differentiation process. Transcript levels of the pluripotency marker *POU5F1* are significantly reduced by day 6 (Figure 2A), while the NEP marker *SOX1* (Figure 2B) and neural stem cell marker nestin (*NES)* (Figure 2C) are both significantly increased at day 6 by four-fold and two-fold, respectively. Transcript levels of the MNP marker oligodendrocyte transcription factor 2 (*OLIG2)* were significantly increased by 11-fold starting at day 12 (D12), which should be the peak time of MNP generation. But, during the differentiation of MNPs into day 18 (D18) early MNs, the expression of *OLIG2* was significantly reduced (Figure 2D). Expression of motor neuron specification marker choline acetyltransferase (CHAT) was not different at the D6 MNP stage, but its levels continued to increase by 3-fold in D12 MNPs and by 5.5-fold in D18 early MNs (Figure 2E). The marker transcript data confirms the generation of NEPs, MNPs, and early MNs while suggesting phenotypic heterogeneity and the presence of a subpopulation of neural stem and progenitor cells even at later time points.

### 3.3. Key Markers of Motor Neuron Differentiation Are Disrupted following Arsenic Exposure

**Figure 3:**
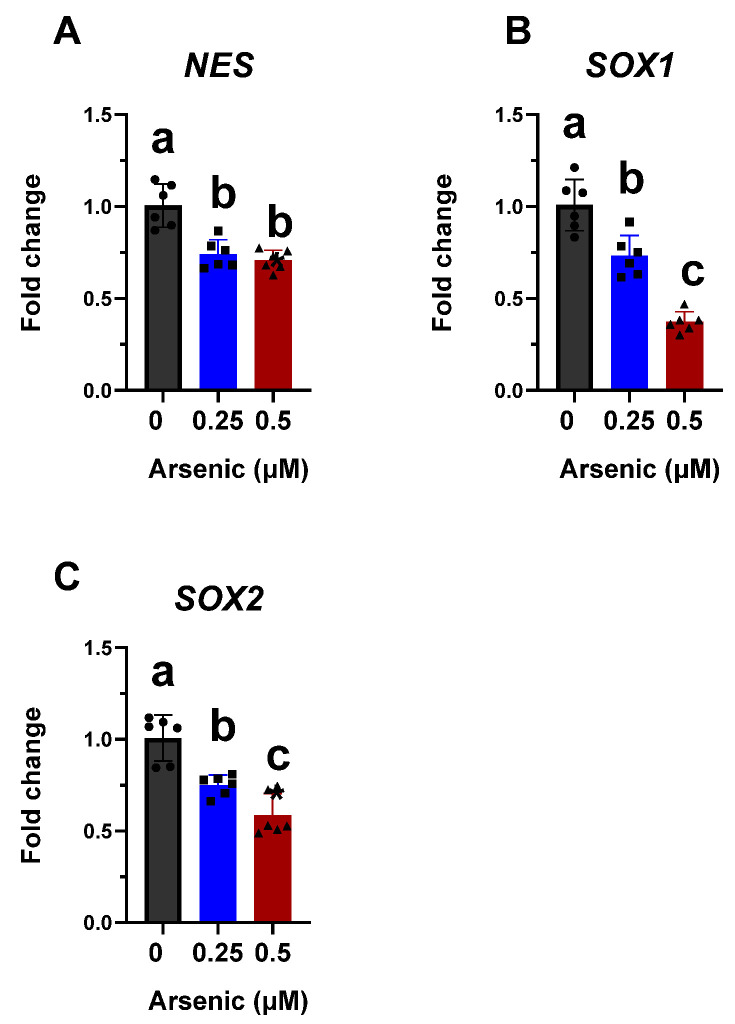
Arsenic exposure reduces neuronal differentiation markers in D6 neuroepithelial progenitor (NEP) cells. Transcript levels of *NES* (**A**), *SOX1* (**B**), and *SOX2* (**C**) were assessed in D6 NEPs exposed to 0 (black, circles), 0.25 (blue, squares), or 0.5 μM (red, triangles) arsenic by qPCR. Fold change was determined using the ΔΔCt method and results were normalized to the geometric mean of Gapdh and β2-microglobulin. Statistical differences were determined using ANOVA followed by Tukey’s multiple comparison test (*p* ≤ 0.05; *n* = 5–6 per exposure group; a = no statistical difference from control; b = statistically different from control; c = statistically different from all other exposure groups).

Previous studies have shown that exposure to low arsenic concentrations inhibits sensory neuron and skeletal muscle differentiation from mouse embryonic stem cells [19,51]. Therefore, we hypothesized that the differentiation into motor neurons, which are anatomically and physiologically connected with skeletal muscle, would also be impaired. To test this hypothesis, human iPS cells were exposed to 0, 0.25, or 0.5 µM arsenic during their differentiation into D6 neuroepithelial progenitors (NEPs) and D12 motor neuron progenitors (MNPs), or to 0 or 0.5 µM arsenic during their differentiation into D18 early and D28 mature motor neurons (MNs). Arsenic treatment does not cause morphological changes in D6 NEPs (Figure 1B). However, transcript levels of neural progenitor/stem cell marker *NES* were significantly decreased in D6 NEPs exposed to 0.25 and 0.5 μM As, by 1.3- and 1.4- fold, respectively (Figure 3A). Similarly, transcript levels of the NEP marker *SOX1* were significantly downregulated by 1.4- and 2.7-fold in NEPs treated with 0.25 and 0.5 μM As, respectively (Figure 3B). *SOX2* transcript levels were also significantly reduced by 1.3- and 1.7-fold in NEPs exposed to 0.25 and 0.5 μM As, respectively (Figure 3C). Previous studies have suggested that *NES* expression positively correlates with Sox2 [52,53]. Furthermore, Sox binding sites have been identified on the nestin enhancer indicating the existence of a regulatory network and of a synergistic interaction between Sox and nestin [54]. There is a positive correlation between *SOX2*, *SOX1,* and *NES* transcript levels in NEPs (Supplementary Table S4).

**Figure 4:**
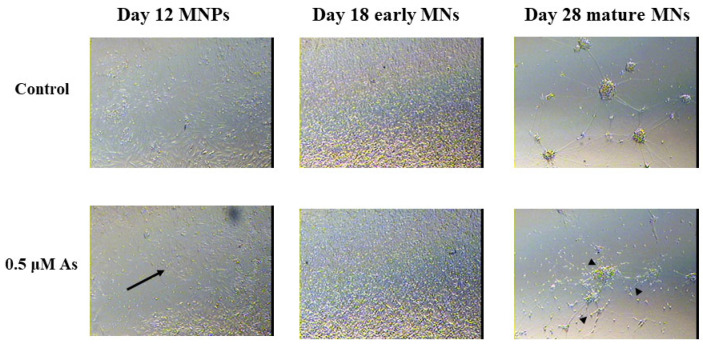
Arsenic exposure leads to morphological alterations of MNPs and MNs. Representative images at 10X of D12 MNPs, D18 early MNs, and at 20X of D28 mature MNs exposed to 0 or 0.5 μM arsenic. The arsenic-exposed D12 MNPs have less concentric organization and fewer elongated MNPs (arrows) while the arsenic-exposed D28 mature MNs have reduced neurite length (arrows heads).

At the next stage of motor neuron formation, we observed morphological differences in 0.5 μM arsenic-treated D12 MNPs, which are less elongated and lose their spatial organization compared with control MNPs (Figure 4).

**Figure 5:**
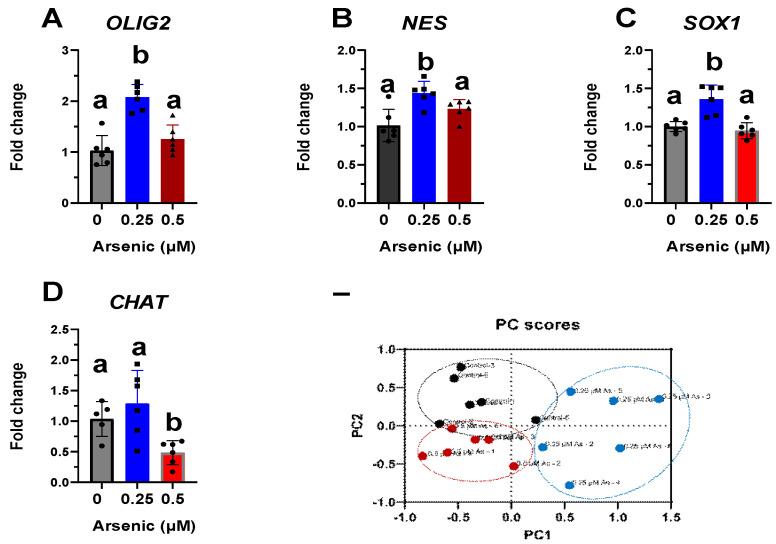
Altered transcript levels of neuronal differentiation markers in D12 motor neuron progenitor (MNP) cells. Expression levels of *OLIG2* (**A**), *NES* (**B**), *SOX1* (**C**), and CHAT (**D**) mRNA were assessed by qPCR in D12 MNPs exposed to 0 (black, circles), 0.25 (blues, squares), or 0.5 μM arsenic (red, triangles). Fold change was determined using the ΔΔCt method and results were normalized to the geometric mean of Gapdh and β2-microglobulin. Statistical differences were determined using ANOVA followed by Tukey’s multiple comparison test (*p* ≤ 0.05; *n* = 5–6 per exposure group; a = no statistical difference from control; b = statistically different from control). Biplot of principal component analysis (PCA) from D12 MNPs exposed to 0, 0.25, or 0.5 μM arsenic (**E**).

**Figure 6:**
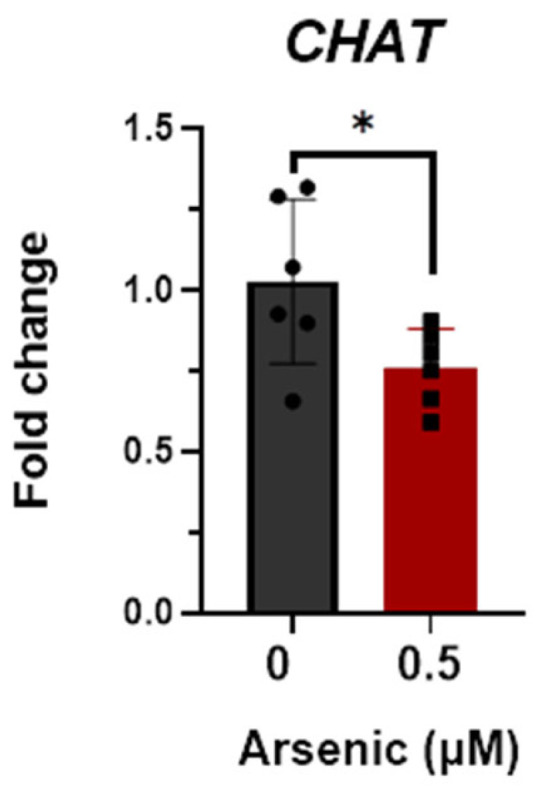
Reductions in choline acetyltransferase (CHAT) mRNA in D18 early motor neurons (MNs). Transcript levels of CHAT were assessed in D18 early MNs exposed to 0 (black, circles) or 0.5 μM arsenic (red, squares) by qPCR. Fold change was determined using the ΔΔCt method and results were normalized to the geometric mean of Gapdh and β2-microglobulin. Statistical differences were determined using Student’s *t*-test (*; *p* ≤ 0.05; *n* = 6 per exposure group).

Transcript levels of *OLIG2* (Figure 5A), *NES* (Figure 5B), and *SOX1* (Figure 5C) are upregulated in the 0.25 μM exposure group but were not altered in the 0.5 μM exposure group compared to the controls. Conversely, transcript levels of CHAT were significantly downregulated in D12 MNPs exposed to 0.5 μM As by 2.1-fold (Figure 5D). These results suggest that at lower arsenic levels, there is a mixed population of cells in which some maintain their stem/neuronal progenitor cell characteristics. Indeed, principal component analyses demonstrate that the three exposure groups are distinct populations of cells, with the 0.25 μM As group showing the widest spread. The two components on the *x*- and *y*-axes explain 96.9% of the total variation (PC1 = 69.1% and PC2 = 27.8%) (Figure 5E). Similar to that of D12 MNPs, CHAT expression was also significantly downregulated by 1.5-fold in early MNs exposed to 0.5 μM As (Figure 6). Taken together, these results indicate that arsenic exposure reduces the differentiation of human iPS cells into motor neurons, leading to altered cellular morphology and neuronal process formation.

### 3.4. Differential Gene Expression Due to Arsenic Exposure in D6 NEPs and D18 Early MNs

**Figure 7:**
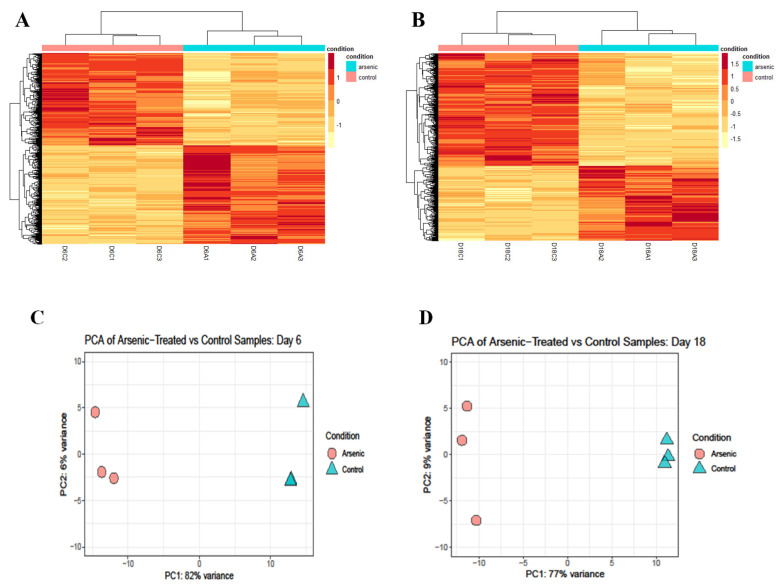

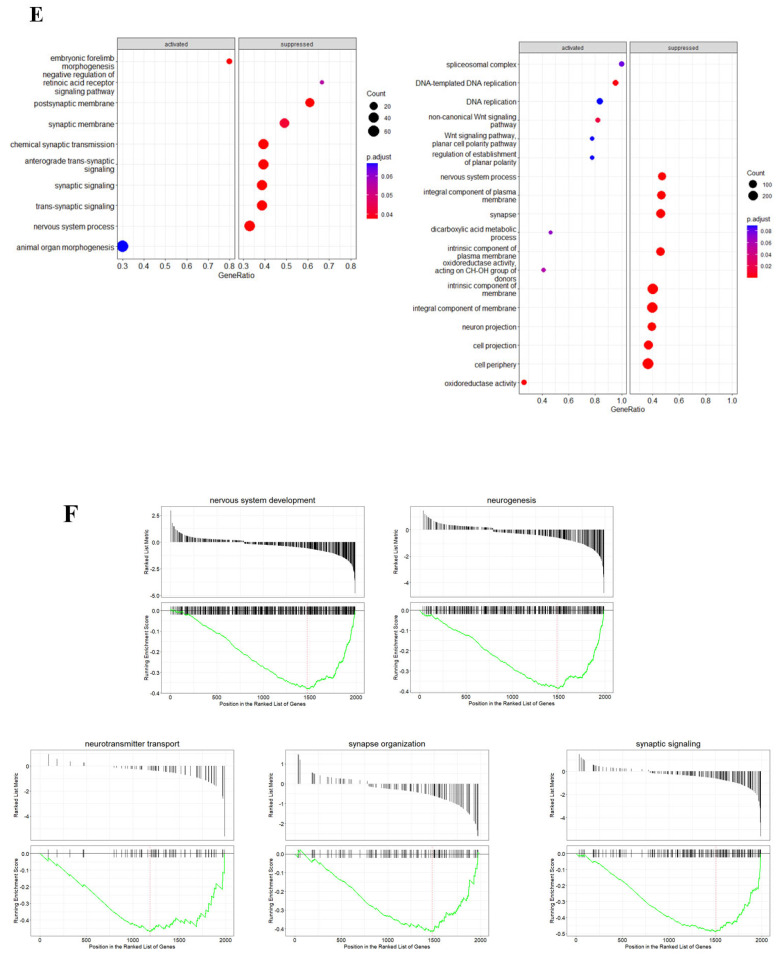
Arsenic exposure drives sample clustering in NEPs and early MNs and disrupts biological processes related to nervous system development and synapses. Heat map of normalized read counts of differentially expressed genes (DEGs) from D6 NEPs (**A**) and D18 early MNs (**B**) exposed to 0 (pink bars) or 0.5 μM arsenic (blue bars). Principal component analysis (PCA) from D6 NEPs (**C**) and D18 early MNs (**D**) exposed to 0 or 0.5 μM arsenic. The distance between the samples represents the differences in gene expression profile (*n* = 3 per exposure group). Dot plot representing gene set enrichment analysis (GSEA) performed on DEGs of arsenic-treated D6 NEPs (**left**) and D18 early MNs (**right**) (**E**). Dot plot shows activated and suppressed GO categories. GSEA performed on DEGs of day 18 early MNs. Gene sets for nervous system development, neurogenesis, neurotransmitter transport, synapse organization, and synaptic signaling were significantly enriched in D18 MNs exposed to arsenic. The vertical black lines present on the *x*-axis represent the genes while the *y*-axis identified the enrichment score. Significance threshold was set at FDR ≤ 0.05 (**F**).

To identify the signaling pathways responsible for the impairment of key markers involved in motor neuron differentiation, we performed RNA sequencing and pathway analysis in D6 NEPs and D18 early MNs. Differential expression analysis suggests that arsenic exposure is driving gene expression changes in both D6 NEPs (Figure 7A) and D18 early MNs (Figure 7B). Principal component analysis (PCA) confirms these results showing that cells exposed to arsenic express different genes than control samples at both time points (Figure 7C,D). For instance, control and arsenic samples are heavily separated along the *x*-axis (PC1), which accounts for 82% of the variation in D6 NEPs (Figure 7C) and 77% of the variation in D18 early MNs (Figure 7D), respectively.

Gene set enrichment analysis (GSEA) indicates that some of the most significantly suppressed categories in arsenic-treated samples were related to synaptic signaling and nervous system process in D6 NEPs, and to synapse, neuron projection, and nervous system processes in D18 early MNs (Figure 7E). Consistently, biological processes in which DEGs presented high enrichment scores were associated with nervous system development, neurogenesis, neurotransmitter transport, synapse organization, and synaptic signaling in D18 early MNs (Figure 7F).

### 3.5. Arsenic Reduces Expression of Genes in Cholinergic Synapses Involved in Acetylcholine Synthesis, Transport, and Degradation

To investigate potential mechanisms disrupted due to arsenic exposure during motor neuron differentiation, pathway analysis was conducted to identify DEGs enriched in KEGG pathways. Our results suggest that neuronal pathways, such as axon guidance (Figure S2A) and neuroactive ligand–receptor interaction (Figure S2B), are disrupted following arsenic exposure in D18 early MNs.

**Figure 8:**
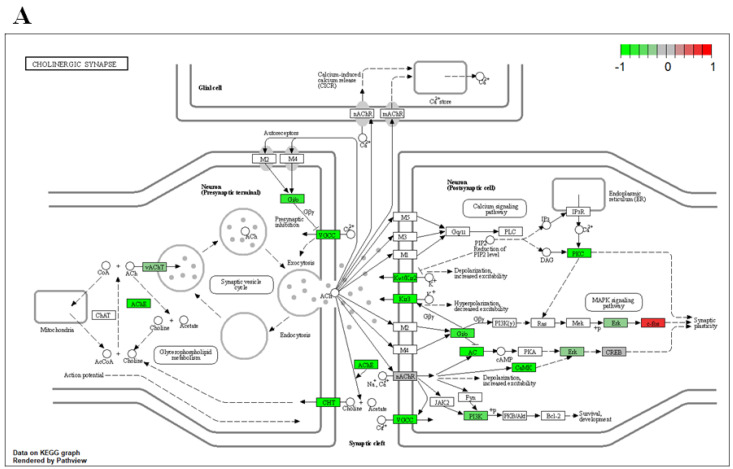

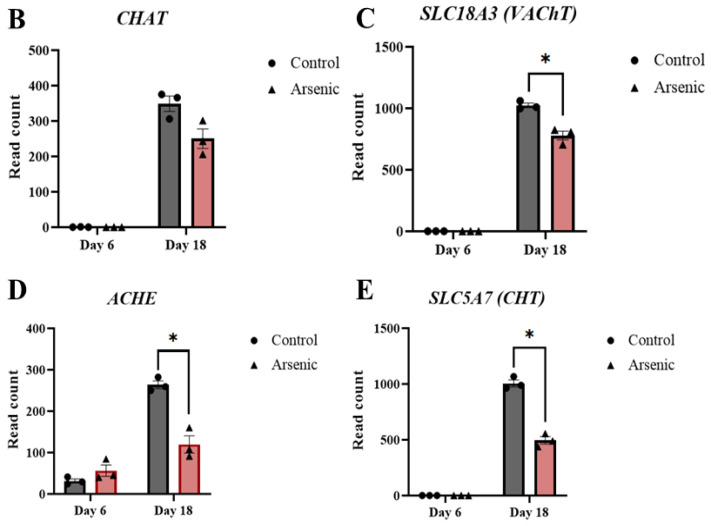
Arsenic exposure downregulates genes involved in acetylcholine synthesis, transport, and degradation of D18 early MNs. KEGG pathway analysis shows several downregulated (green) genes of the cholinergic synapse pathway in arsenic-exposed D18 early MNs (**A**). RNA sequencing normalized read counts of CHAT (**B**), *SLC18A3* (**C**), *ACHE* (**D**), and *SLC5A7* (**E**) from D6 NEPs and D18 early MNs. Data are presented as read count ± SE. Statistical differences (*) were determined by Student’s *t*-test using the adjusted *p*-value (FDR ≤ 0.05).

Mammalian motor neurons can release both acetylcholine and glutamate [55]. Typically upper motor neurons form glutamatergic synapses while lower motor neurons are cholinergic neurons [56]. Interestingly, both pathways associated with glutamatergic synapses (Figure S2C) and cholinergic synapses (Figure 8A) were downregulated in arsenic-treated D18 early MNs. In cholinergic neurons, ChAT is the enzyme involved in the biosynthesis of the neurotransmitter acetylcholine, which is loaded into presynaptic vesicles by the vesicular acetylcholine transporter (VAChT) and released into the synaptic cleft. Afterward, acetylcholine is hydrolyzed by acetylcholinesterase (AChE) into acetate and choline, and choline is transported back into the presynaptic nerve terminal by the choline transporter (ChT). Transcript expression of the cholinergic pathway genes CHAT, *SLC18A3* (encoding for VAChT), *ACHE,* and *SLC5A7* (encoding for ChT) were downregulated in arsenic-treated D18 early MNs by 1.4-, 2-, 2.2-, and 1.3-fold, respectively (Figure 8B–E). Read counts of these genes were consistently increased in D18 early MNs as compared to D6 NEPs, confirming the formation of cholinergic neurons during the differentiation process (Figure 8B–E). These data, coupled with the observed morphological changes, suggest that arsenic exposure may disrupt the proper formation and function of acetylcholine in motor neurons.

### 3.6. Arsenic Exposure Downregulates Protein Expression of MAP2 and ChAT, and Reduces Neurite Length in D28 Mature MNs

**Figure 9:**
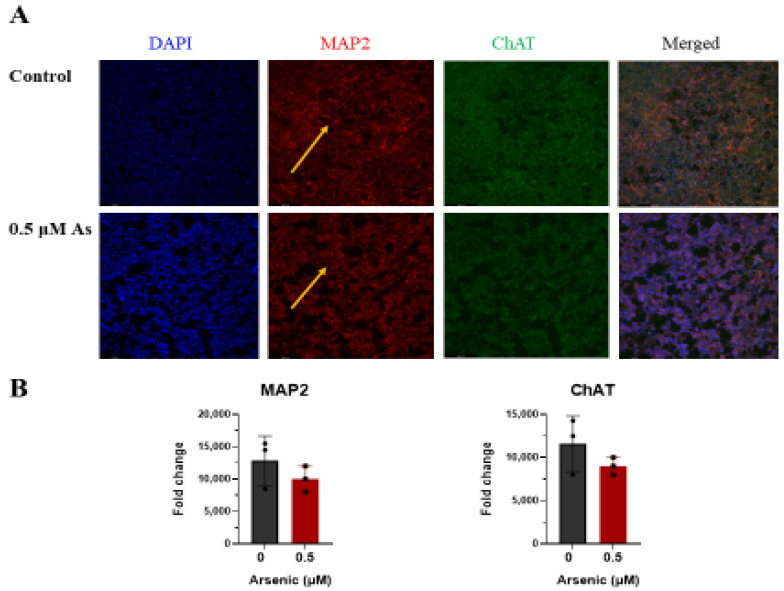
Arsenic exposure alters MAP2 pattern in day 18 early MNs. (**A**) Representative images of MAP2 (red) and ChAT (green) in control and 0.5 μM arsenic-exposed day 18 early MNs. Yellow arrows show differences in MAP2 pattern. (**B**) Relative fluorescence of MAP2 (left) and ChAT (right) was determined in ImageJ and is presented as integrated density value (IDV) ± SE between control (black) and 0.5 μM arsenic (red) (*n* = 3 per exposure group). Statistical differences were determined using Student’s *t*-test (*; *p* ≤ 0.05).

**Figure 10:**
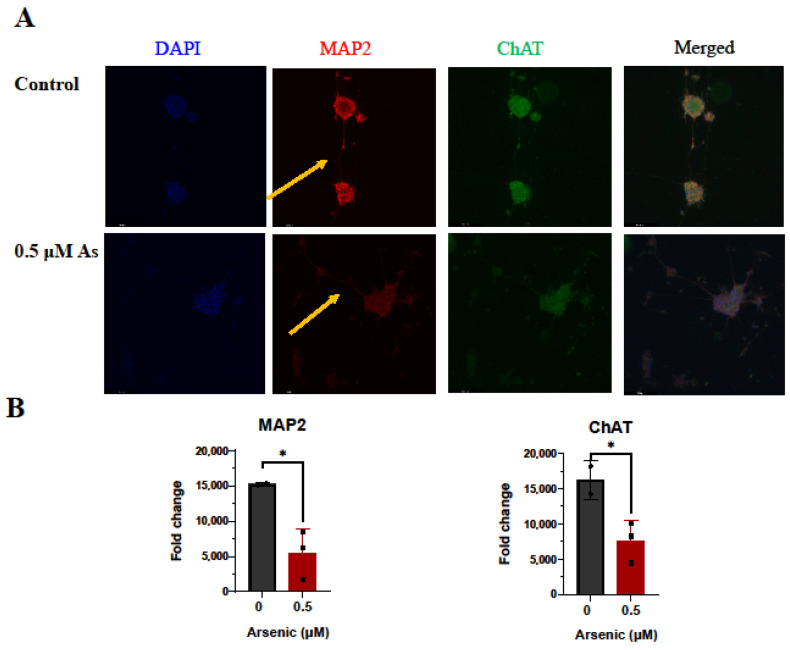

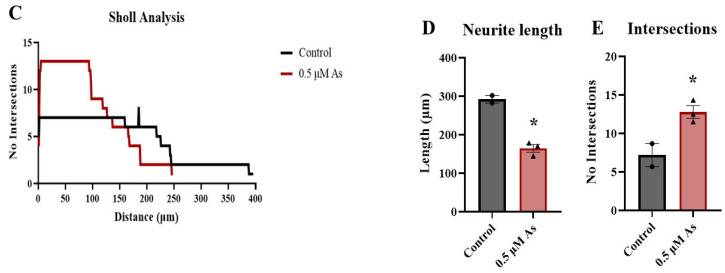
Arsenic exposure reduces neurite length and downregulates protein levels of MAP2 and ChAT in day 28 mature MNs. (**A**) Representative images of MAP2 (red) and ChAT (green) in control and 0.5 μM arsenic-exposed day 28 mature MNs. Arrows show differences in neurite length. (**B**) Relative fluorescence of MAP2 (**left**) and ChAT (**right**) in the cell body was determined in ImageJ. Protein expression was assessed in two to six cells per biological replicate (*n* = 3 replicates per exposure group), and data are presented as integrated density value (IDV) ± SE for control (black, circles) and 0.5 μM arsenic (red, triangles). Statistical differences were determined using Student’s *t*-test (*; *p* ≤ 0.05). (**C**) Sholl analysis of neural processes of MAP2^+^ day 28 mature MNs was performed on one to two cells per biological replicate (*n* = 3 replicates per exposure group). (**D**) Average neurite length of neuronal processes of MAP2^+^ day 28 mature MNs. Analysis was performed on *n* = 3 per exposure group and one to two cells per biological replicate. (**E**) Average number of intersections obtained from C.

The effects of arsenic exposure on protein expression of the neuronal marker MAP2 [57,58], and cholinergic marker ChAT were investigated by immunohistochemistry in D18 early motor neurons. While no differences in MAP2 and ChAT protein expression were noted, fewer MAP2+ neuronal extensions can be seen in arsenic-exposed D18 early MNs (arrows; Figure 9A,B). However, in D28 mature MNs, protein expression of MAP2 and ChAT in the cell body of was significantly downregulated in arsenic-exposed samples by 2.8- and 2.1-fold, respectively (Figure 10A,B).

Moreover, morphological differences were observed in arsenic-treated MAP2+ neurites, which are visually less elongated (Figure 10A). Sholl analysis confirmed that arsenic significantly reduces neurite length by 1.8 -fold while increasing the number of intersections (Figure 10C–E).

### 3.7. Arsenic Exposure In Vivo Downregulated ChAT Protein Expression in Adult Mice Hippocampi

To examine the in vivo effects of arsenic exposure on ChAT and MAP2, we evaluated their expression in the hippocampi of adult male mice exposed to 0 or 100 ppb arsenic for five weeks. Cholinergic neurons are distributed in the hippocampus as acetylcholine modulates hippocampal-dependent memory function and learning [59,60]. Immunohistochemical analysis confirms the presence of MAP2+ neurofilaments and ChAT+ cholinergic neurons in hippocampi collected from adult mice. While no differences were observed in the protein expression of MAP2, arsenic treatment significantly reduced hippocampal ChAT levels (Figure S3).

## 4. Discussion

The results of this study demonstrate that stage-specific motor neuron differentiation markers, such as *SOX1*, *NES*, *OLIG2,* and CHAT, are dysregulated due to arsenic exposure. Further, expression of genes in mature neurons involved in the acetylcholine cycle such as CHAT, *SLC18A3* (encoding for VAChT), *SLC5A7* (encoding for ChT), and *ACHE* are also reduced. Arsenic exposure also downregulates ChAT protein expression in mature MNs while reducing neurite length, suggesting that arsenic might impair the proper function of the cholinergic synapse.

### 4.1. Arsenic Impairs Transcript Levels of Key Motor Neuron Differentiation Markers

Species-specific responses in the metabolism and whole-body retention of arsenic have been reported. For instance, humans are more susceptible to arsenic than mice as they have reduced levels of arsenic methyltransferase (As3MT), which leads to increased body retention [61,62]. To our knowledge, few studies have used human iPS cells as in vitro models to investigate the effects of arsenic exposure on cellular differentiation. Neuronal differentiation impairment following arsenic exposure has been previously reported in P19 mouse embryonic stem cells [19,51,63], and mouse neuronal N2a and rat PC12 cells [17,18,64]. Recently, investigators exposed 50 μM arsenic for 48 h to neural progenitor cells derived from human iPS cells [65], and found neurite damage. However, the current study is the first to assess the effects of lower, more environmentally relevant arsenic concentrations on the cellular differentiation of human iPS cells-derived motor neurons.

During motor neuron formation, there are several well-studied markers that can be used to follow the time course of differentiation, including *SOX1*, *NES*, *OLIG2,* and CHAT. The transcription factor Sox1 is the earliest and most specific marker for neuroepithelial cells, as it is expressed by progenitor cells in the nervous system during fetal neurodevelopment [66,67]. Nestin is an intermediate filament protein, encoded by the *NES* gene, which is highly expressed in multipotent stem cells and neural progenitor cells of the developing brain [68,69]. Nestin was first described as a neuroepithelial stem cell marker and it is commonly used to identify progenitor populations [70]. Together, Sox1 and nestin represent early markers of neural stem cells [71]. Our findings show that transcript levels of both *SOX1* and *NES* are significantly downregulated in day 6 NEPs in a dose-dependent manner, while day 12 MNPs have increased expression in the 0.25 μM exposure group. These results may imply that the 0.5 μM exposure group remains even further behind in their differentiation to motor neurons. Indeed, the principal component analysis (PCA) indicates that each of the three day 12 exposure groups form its own distinct population of cells. To our knowledge, this is the first study to show that arsenic exposure disrupts the early stages of motor neuron differentiation in human iPS cells.

### 4.2. Arsenic Downregulates ChAT Expression and Inhibits Genes Involved in Acetylcholine Transport and Degradation

One of the distinguishing features of motor neurons is their ability to synthesize and release acetylcholine (ACh) as a neurotransmitter. Choline acetyltransferase (ChAT) is the enzyme responsible for the biosynthesis of acetylcholine by transferring an acetyl group from acetyl-CoA to choline (reviewed in [72]). It is considered a specific marker for cholinergic neurons [73,74], and indeed, CHAT expression was significantly downregulated by 1.5- to 2-fold in both D12 MNPs and D18 early MNs following arsenic exposure. Following acetylcholine synthesis, the neurotransmitter is then loaded by the vesicular acetylcholine transporter (VAChT; encoded by *SLC18A3*) into presynaptic vesicles and stored until it is released in the synaptic cleft [75]. After release into the synapse, acetylcholine is hydrolyzed into choline and acetate by the enzyme acetylcholinesterase (AChE) to terminate neuronal signaling. Lastly, the high-affinity choline transporter (ChT; encoded by *SLC5A7*) is responsible for the choline re-uptake into the cholinergic neuron [76]. RNA sequencing of the D18 early motor neurons followed by KEGG analysis further illustrates that the cholinergic synapse pathway is impaired. Specifically, read counts of CHAT, *SLC18A3*, *ACHE,* and *SLC5A7* were all significantly reduced by 1.3- to 2.4-fold in D18 early motor neurons following exposure to 0.5 μM arsenic. Read counts for MAP2 in D18 early MNs were not altered, meaning that the number of neurons did not change.

A high density of cholinergic neurons has been reported in the cerebral cortex and the hippocampus [77], where they play a role in synaptic transmission, plasticity, and neuronal network formation [73]. Additionally, cholinergic transmission is critical for many cognitive functions including memory and learning [77,78]. A recent study has suggested that perinatal arsenic exposure leads to cholinergic system impairment in the brain of developing rats [29]. Specifically, there was a significant decrease in AChE activity and ChAT protein levels in the frontal cortex and the hippocampus of rats perinatally exposed to sodium arsenite. Similarly, AChE activity in the hippocampus and frontal cortex was reduced, along with reduced ChAT protein expression, in adult rats exposed to sodium arsenite for four weeks [27]. These in vivo findings are consistent with our results that showed reduced ChAT levels in the hippocampi of mice exposed to 100 ppb arsenic for five weeks. As with the D18 early motor neurons, the amount of MAP2 protein was not altered, indicating that arsenic exposure at these concentrations is altering cholinergic neurons, rather than causing general neuronal toxicity.

### 4.3. Arsenic Reduces Neurite Length in Mature MNs

When taking the cultures out to 28 days to form mature motor neurons, ChAT mRNA and protein expression are also reduced following exposure to 0.5 μM arsenic. The neurites from arsenic-exposed motor neurons appear to be less elongated than their control counterparts. Indeed, neurite tracing and Sholl analysis demonstrate the length of MAP2+ neurites was reduced. Several studies conducted in vivo and in vitro have also reported decreased neurite length following arsenic exposure. For instance, neurite length was significantly reduced by 1 μM sodium arsenite in primary cultured neurons [21], by 5 μM sodium arsenite and 0.5 μM arsenic trioxide in Neuro-2a cells [18,20], and by 5 μM sodium arsenite in differentiated neurons derived from mouse embryonic forebrains [79]. Similarly, treatment with sodium arsenite caused a dose-dependent inhibition of neurite length in differentiated human neuroblastoma SH-SY5Y cells [22]. Additionally, neurite length reduction has been observed in mice following prenatal exposure to sodium arsenite from gestational day 8 to 18 [80]. MNs are characterized by long axons that connect them to skeletal muscle [56] and, importantly, loss and degeneration of MNs can cause motor neuron diseases including amyotrophic lateral sclerosis and spinal muscular atrophy [81,82]. Arsenic-induced inhibition of neurite length could also lead to improper function of neuronal circuits as impairment in the patterns and length of neuronal processes has been linked to cognitive alterations and mental retardation [80].

## 5. Conclusions

Exposure to 0.5 µM arsenic during motor neuron differentiation impaired the cholinergic pathway. Downregulation of genes involved in acetylcholine synthesis (CHAT), transport (*SLC18A3* and *SLC5A7*), and degradation (*ACHE*) along with reduced ChAT protein expression and neurite outgrowth suggest that proper function of cholinergic neurons might be impaired following arsenic exposure. Taken together, these results suggest that arsenic-induced impairment of cholinergic functions could be responsible for the memory and learning deficits along with the locomotor and neurological alterations reported by epidemiological and in vivo studies.

## Data Availability

Data will be made available upon reasonable request.

## Supplementary Materials

The following supporting information can be downloaded at: https://www.mdpi.com/article/10.3390/toxics11080644/s1, Table S1: Supplements and growth factors used during differentiation of human iPS cells into motor neurons. Table S2: Primer sequences of genes quantified by qPCR. Table S3: Correlation between transcript levels of *SOX2*, *POU5F1* and *NANOG* in human iPS cells exposed to 0, 0.1 or 0.5 μM arsenic for six days. Table S4: Correlation between transcript levels of *SOX2*, *SOX1* and *NES* in day 6 NEPs exposed to 0, 0.25, 0.5 and 0.75 μM. Figure S1: Arsenic increases transcript levels of pluripotency markers *SOX2*, *POU5F1* and *NANOG* in human iPS cells. Transcript levels of *SOX2* (A), *POU5F1* (B) and *NANOG* (C) were assessed by qPCR in human iPS cells exposed to 0, 0.1 or 0.5 μM arsenic for six days. Fold change was determined using the ddCt method and results were normalized to the geometric mean of Gapdh and β2-microglobulin. Statistical differences were determined using ANOVA followed by Tukey’s multiple comparison test (*; *p* ≤ 0.05) (n = 5–6 per exposure group). Figure S2: Arsenic exposure impairs glutamatergic synapse, axon guidance and neuroactive ligand-receptor interaction pathways. KEGG pathway analysis of pathways regulating axon guidance (A), neuroactive ligand-receptor interaction pathway (B) and glutamatergic synapse pathway (C) shows various downregulated (green) genes and a few upregulated (red) genes in arsenic-exposed day 18 early MNs. Figure S3: Arsenic exposure impairs ChAT protein levels in adult mice hippocampi. (A) Representative images of 3D z-stacked images of MAP2 (yellow) and ChAT (blue) in hippocampi harvested from adult mice exposed to 100 ppb of arsenic for five weeks. (B) Relative fluorescence of MAP2 (left) and ChAT (right) was determined in ImageJ and is presented as integrated density value (IDV) ± SE (n = 3–4 per exposure group). Statistical differences were determined using Student’s *t*-test (*; *p* ≤ 0.05).
