# Supplementary material for: Arsenic Impairs Differentiation of Human Induced Pluripotent Stem Cells into Cholinergic Motor Neurons
Source: Toxics. 2023 Jul 25;11(8):644. doi: 10.3390/toxics11080644 (PMC10458826; doi:10.3390/toxics11080644)

Supplemental Data

Supplementary Table S1. Supplements and growth factors used during differentiation of human iPS cells into motor neurons

**Human iPS cells to NEPs (day 0 to day 6)**			
**Growth factor**	**Concentration**	**Role**	**Source**
CHIR99021	3 μM	Wnt signaling activator	StemCell
SB431542	2 μM	TGF-β/Activin/NODAL signaling inhibitor	Sigma Aldrich
DMH-1	2 μM	BMP signaling inhibitor	Tocris
**NEPs to MNPs (day 6 to day 12)**			
**Growth factor**	**Concentration**	**Role**	**Source**
CHIR99021	1 μM	Wnt signaling activator	StemCell
SB431542	2 μM	TGF-β/Activin/NODAL signaling inhibitor	Sigma Aldrich
DMH-1	2 μM	BMP signaling inhibitor	Tocris
Retinoic acid	0.1 μM	Nuclear receptors RARs activator	Acros Organics
Purmorphamine	0.5 μM	SHH signaling agonist	Millipore Sigma
**MNPs to early MNs (day 12 to day 18)**			
**Growth factor**	**Concentration**	**Role**	**Source**
Retinoic acid	0.5 μM	Nuclear receptors RARs activator	Acros Organics
Purmorphamine	0.1 μM	SHH signaling agonist	Millipore Sigma
**Early MNs to mature MNs (day 18 to day 28)**			

**Growth factor**	**Concentration**	**Role**	**Source**
Retinoic acid	0.5 µM	Nuclear receptors RARs activator	Acros Organics
Purmorphamine	0.1 µM	SHH signaling agonist	Millipore Sigma
Compound E	0.1 µM	Notch signaling inhibitor	Millipore Sigma
IGF-1	10 ng/ml	PI3K/Akt and Ras/ERK pathway activator	Sigma Aldrich
BNDF	10 ng/ml	TrkB tyrosine kinase receptor activator	R&D systems
CNTF	10 ng/ml	Neurotrophic and survival factor for embryonic motor neurons	R&D systems

Supplementary Table S2. Primer sequences of genes quantified by qPCR

Gene Name	Primer Sequence	
	Forward (5‘-3’)	Reverse (5’-3’)
GAPDH	CCCTTCATTGACCTCAACTACA	ATGACAAGCTTCCCGTTCTC
β2-MICROGLOBULIN	CCAGCGTACTCCAAAGATTCA	TGGATGAAACCCAGACACATAG
SOX2	GCTACAGCATGATGCAGGACCA	TCTGCGAGCTGGTCATGGAGTT
POU5F14	CCTGAAGCAGAAGAGGATCAC	AGATGGTCGTTTGGCTGAATA
NANOG	TGAAATCTAAGAGGTGGCAGAA	CCTGGTGGTAGGAAGAGTAAAG
SOX1	AATGTAGTAAGGCAGGTCCAAG	GGTGGTGGTGGTAATCTCTTT
NES	CACTTCAGTTTAGAGGCTAAGG	CCCTCTATGGCTGTTTCTTTCT
OLIG2	CAGAAGCGCTGATGGTCATA	CTCCCAAATCAACGAGAGACA
CHAT	GGAGTAAGAAAGCAACCAGAGA	CACAAAGAAGGGAGACCTACAG

Supplementary Table S3. Correlation between transcript levels of *SOX2*, *POU5F1* and *NANOG* in human iPS cells exposed to 0, 0.1 or 0.5 µM arsenic for six days.

**Correlation**			
	SOX2	***POU5F1***	***NANOG***
SOX2	1	0.94	0.96
POU5F1	0.94	1	0.99
NANOG	0.96	0.99	1

Supplementary Table S4. Correlation between transcript levels of *SOX2*, *SOX1* and *NES* in day 6 NEPs exposed to 0, 0.25, 0.5 and 0.75 µM.

**Correlation**			
	SOX1	***SOX2***	***NES***
SOX1	1	0.78	0.78
SOX2	0.78	1	0.76
NES	0.78	0.76	1

Supplementary Figure S1. Arsenic increases transcript levels of pluripotency markers *SOX2*, *POU5F1* and *NANOG* in human iPS cells. Transcript levels of *SOX2* (A), *POU5F1* (B) and *NANOG* (C) were assessed by qPCR in human iPS cells exposed to 0, 0.1 or 0.5 µM arsenic for six days. Fold change was determined using the ddCt method and results were normalized to the geometric mean of Gapdh and β2-microglobulin. Statistical differences were determined using ANOVA followed by Tukey's multiple comparison test (*; $p \leq 0.05$) (n = 5-6 per exposure group).

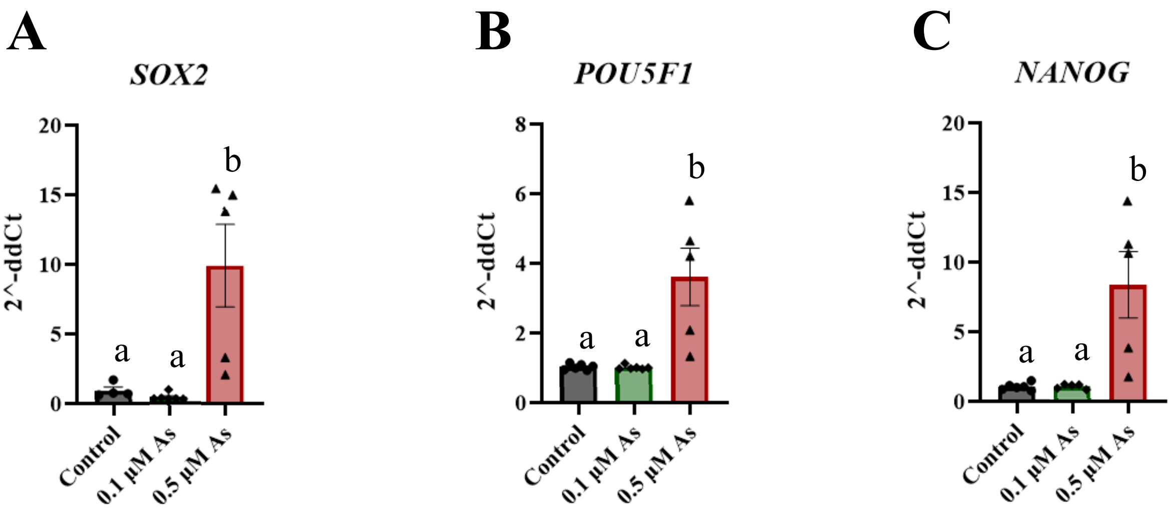

Supplementary Figure S2. Arsenic exposure impairs glutamatergic synapse, axon guidance and neuroactive ligand-receptor interaction pathways. KEGG pathway analysis of pathways regulating axon guidance (A), neuroactive ligand-receptor interaction pathway (B) and glutamatergic synapse pathway (C) shows various downregulated (green) genes and a few upregulated (red) genes in arsenic-exposed day 18 early MNs.

A

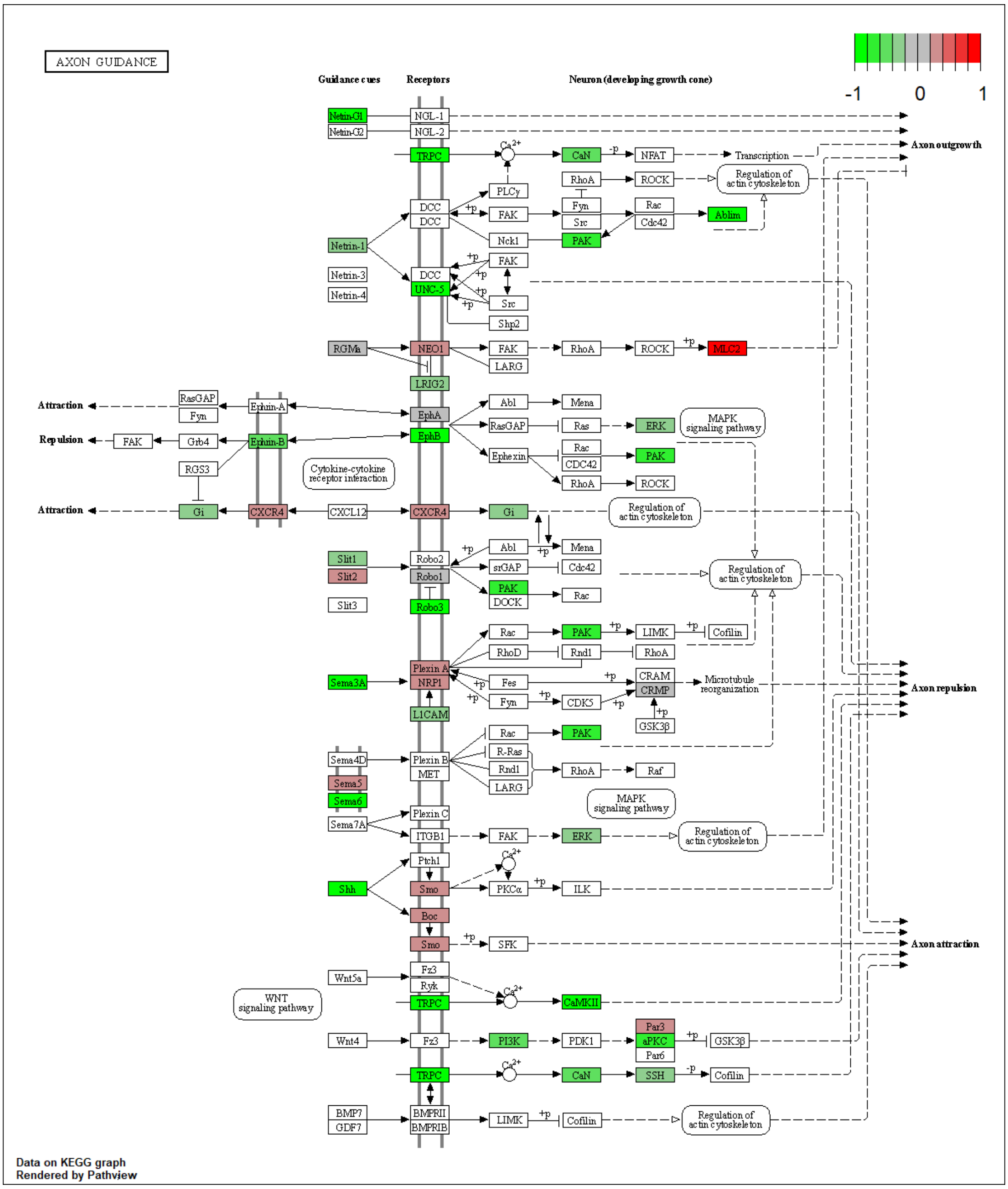

B

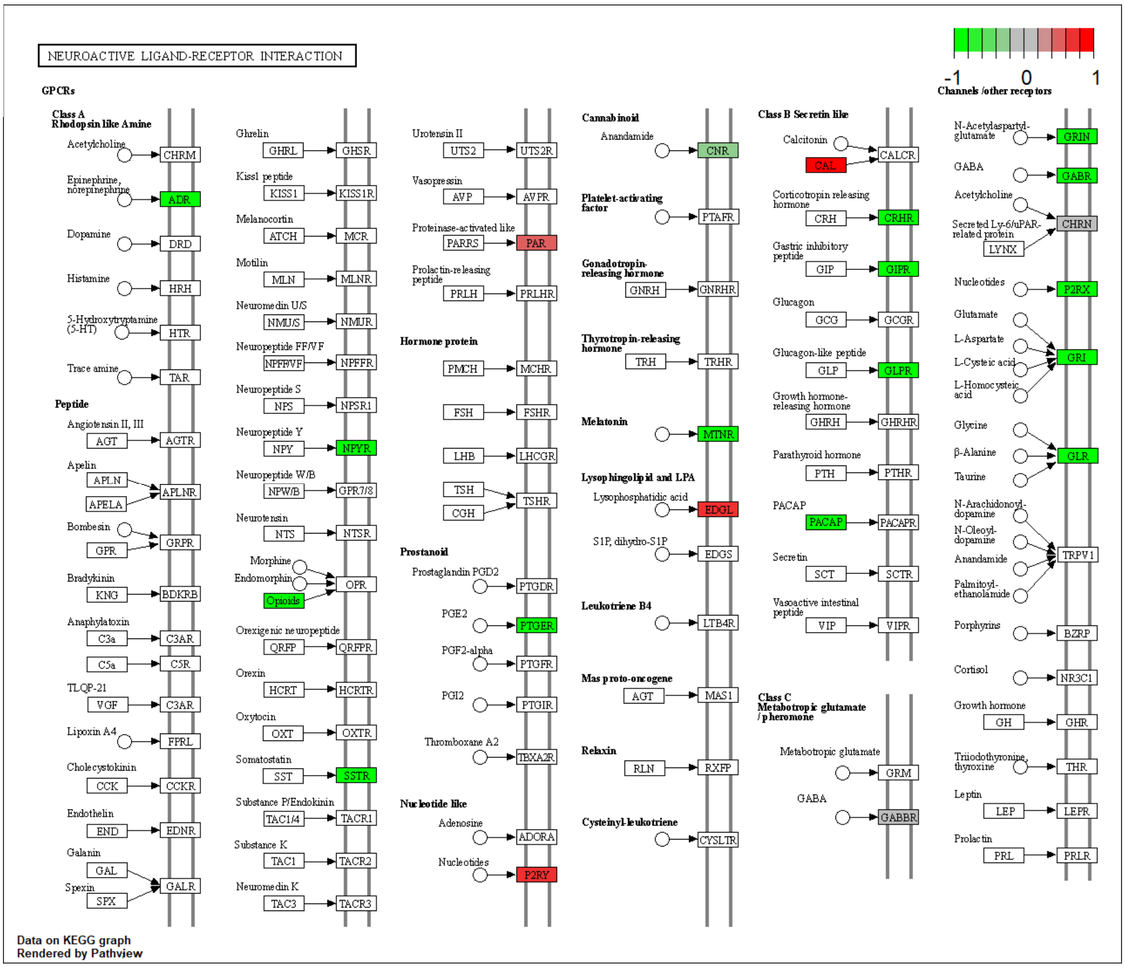
NEUROACTIVE LIGAND-RECEPTOR INTERACTION
-1 0 1
Channels /other receptors
GPCRs
Class A
Rhodopsin like Amine
Acetylcholine
CHRM
Epinephrine, norepinephrine
ADR
Dopamine
DRD
Histamine
HRH
5-Hydroxytryptamine (5-HT)
HTR
Trace amine
TAR
Peptide
Angiotensin II, III
AGT
AGTR
Apelin
APLN
APELA
APLNR
Bombesin
GPR
GRPR
Bradykinin
KNG
BDKRB
Anaphylatoxin
C3a
C3AR
C5a
C5R
TLQP-21
VGF
C3AR
Lipoxin A4
FPRL
Cholecystokinin
CCK
CCKR
Endothelin
END
EDNR
Galanin
GAL
Spexin
SPX
GALR
Ghrelin
GHRL
GHSR
Kiss1 peptide
KISS1
KISS1R
Melanocortin
ATCH
MCR
Motilin
MLN
MLNR
Neuromedin U/S
NMU/S
NMUR
Neuropeptide FF/VF
NPFF/VF
NPFFR
Neuropeptide S
NPS
NPSR1
Neuropeptide Y
NPY
NPYR
Neuropeptide W/B
NPW/B
GPR7/8
Neurotensin
NTS
NTSR
Morphine
Endomorphin
Opioids
OPR
Orexigenic neuropeptide
QRFP
QRFPR
Orexin
HCRT
HCRTR
Oxytocin
OXT
OXTR
Somatostatin
SST
SSTR
Substance P/Endokinin
TAC1/4
TACR1
Substance K
TAC1
TACR2
Neuromedin K
TAC3
TACR3
Urotensin II
UTS2
UTS2R
Vasopressin
AVP
AVPR
Proteinase-activated like
PARRS
PAR
Prolactin-releasing peptide
PRLH
PRLHR
Hormone protein
PMCH
MCHR
FSH
FSHR
LHB
LHCGR
TSH
CGH
TSHR
Prostanoid
Prostaglandin PGD2
PTGDR
PGE2
PTGER
PGF2-alpha
PTGFR
PGI2
PTGIR
Thromboxane A2
TBXA2R
Nucleotide like
Adenosine
ADORA
Nucleotides
P2RY
Cannabinoid
Anandamide
CNR
Platelet-activating factor
PTAFR
Gonadotropin-releasing hormone
GNRH
GNRHR
Thyrotropin-releasing hormone
TRH
TRHR
Melatonin
MTNR
Lysophingolipid and LPA
Lysophosphatidic acid
EDGL
S1P, dihydro-S1P
EDGS
Leukotriene B4
LTB4R
Mas proto-oncogene
AGT
MAS1
Relaxin
RLN
RXFP
Cysteinyl-leukotriene
CYSLTR
Class B Secretin like
Calcitonin
CAL
CALCR
Corticotropin releasing hormone
CRH
CRHR
Gastric inhibitory peptide
GIP
GIPR
Glucagon
GCG
GCGR
Glucagon-like peptide
GLP
GLPR
Growth hormone-releasing hormone
GHRH
GHRHR
Parathyroid hormone
PTH
PTHR
PACAP
PACAP
PACAPR
Secretin
SCT
SCTR
Vasoactive intestinal peptide
VIP
VIPR
Class C
Metabotropic glutamate /pheromone
Metabotropic glutamate
GRM
GABA
GABBR
N-Acetylaspartyl-glutamate
GRIN
GABA
GABR
Acetylcholine
Secreted Ly-6/uPAR-related protein
LYNX
CHRN
Nucleotides
P2RX
Glutamate
L-Aspartate
L-Cysteic acid
L-Homocysteic acid
GRI
Glycine
β-Alanine
Taurine
GLR
N-Arachidonoyl-dopamine
N-Oleoyl-dopamine
Anandamide
Palmitoyl-ethanolamide
TRPV1
Porphyrins
BZRP
Cortisol
NR3C1
Growth hormone
GH
GHR
Triiodothyronine, thyroxine
THR
Leptin
LEP
LEPR
Prolactin
PRL
PRLR
Data on KEGG graph
Rendered by Pathview

C

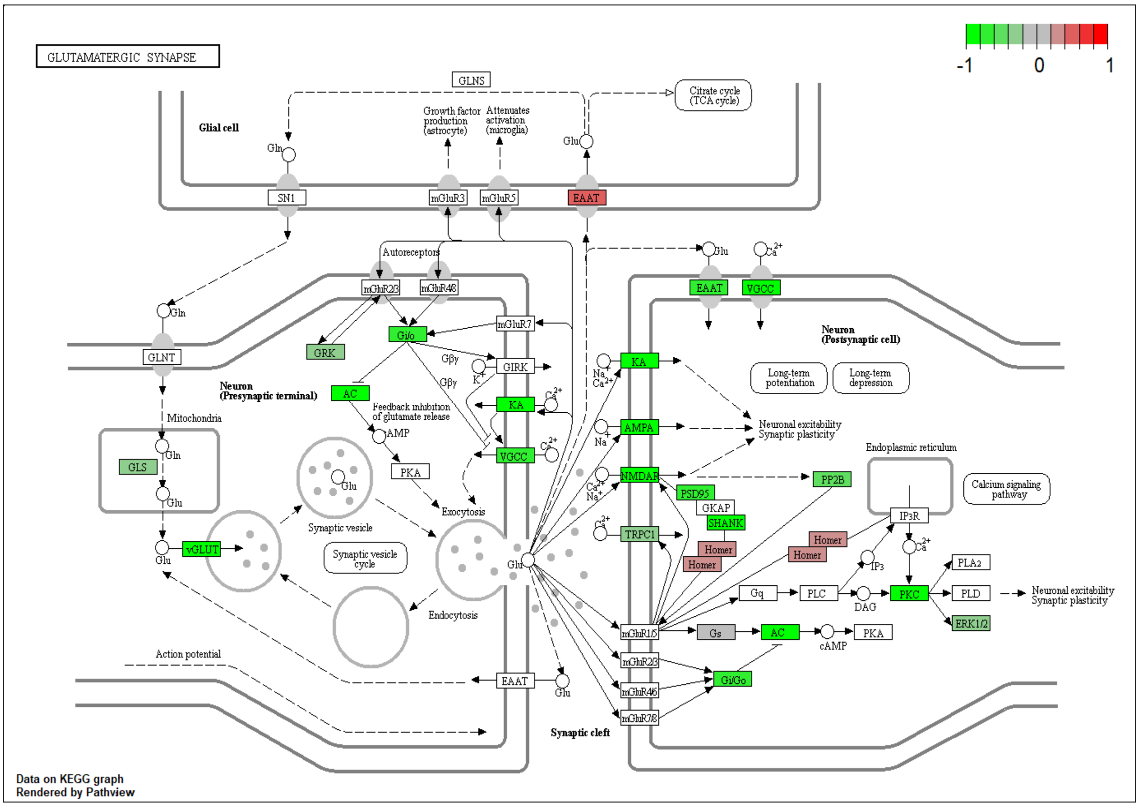
GLUTAMATERGIC SYNAPSE
-1
0
1
GLNS
Citrate cycle (TCA cycle)
Glial cell
Growth factor production (astrocyte)
Attenuates activation (microglia)
Gln
Glu
SN1
mGluR3
mGluR5
EAAT
Autoreceptors
mGluR2/3
mGluR4/8
mGluR7
Gi/o
GRK
GLNT
Neuron (Presynaptic terminal)
AC
Gβγ
GIRK
KA
VGCC
Feedback inhibition of glutamate release
AMP
PKA
Mitochondria
GLS
vGLUT
Synaptic vesicle
Synaptic vesicle cycle
Exocytosis
Endocytosis
Action potential
Synaptic cleft
Neuron (Postsynaptic cell)
Long-term potentiation
Long-term depression
AMPA
Neuronal excitability Synaptic plasticity
NMDAR
PSD95
GKAP
SHANK
Homer
PP2B
Endoplasmic reticulum
Calcium signaling pathway
IP3R
TRPC1
IP3
Gq
PLC
DAG
PKC
PLA2
PLD
ERK1/2
mGluR1/5
Gs
cAMP
mGluR2/3
mGluR4/6
mGluR7/8
Gi/Go
Data on KEGG graph
Rendered by Pathview

Supplementary Figure S3. Arsenic exposure impairs ChAT protein levels in adult mice hippocampi. (A) Representative images of 3D z-stacked images of MAP2 (yellow) and ChAT (blue) in hippocampi harvested from adult mice exposed to 100 ppb of arsenic for five weeks. (B) Relative fluorescence of MAP2 (left) and ChAT (right) was determined in ImageJ and is presented as integrated density value (IDV) ± SE (n = 3-4 per exposure group). Statistical differences were determined using Student's t-test (*; $p \leq 0.05$).

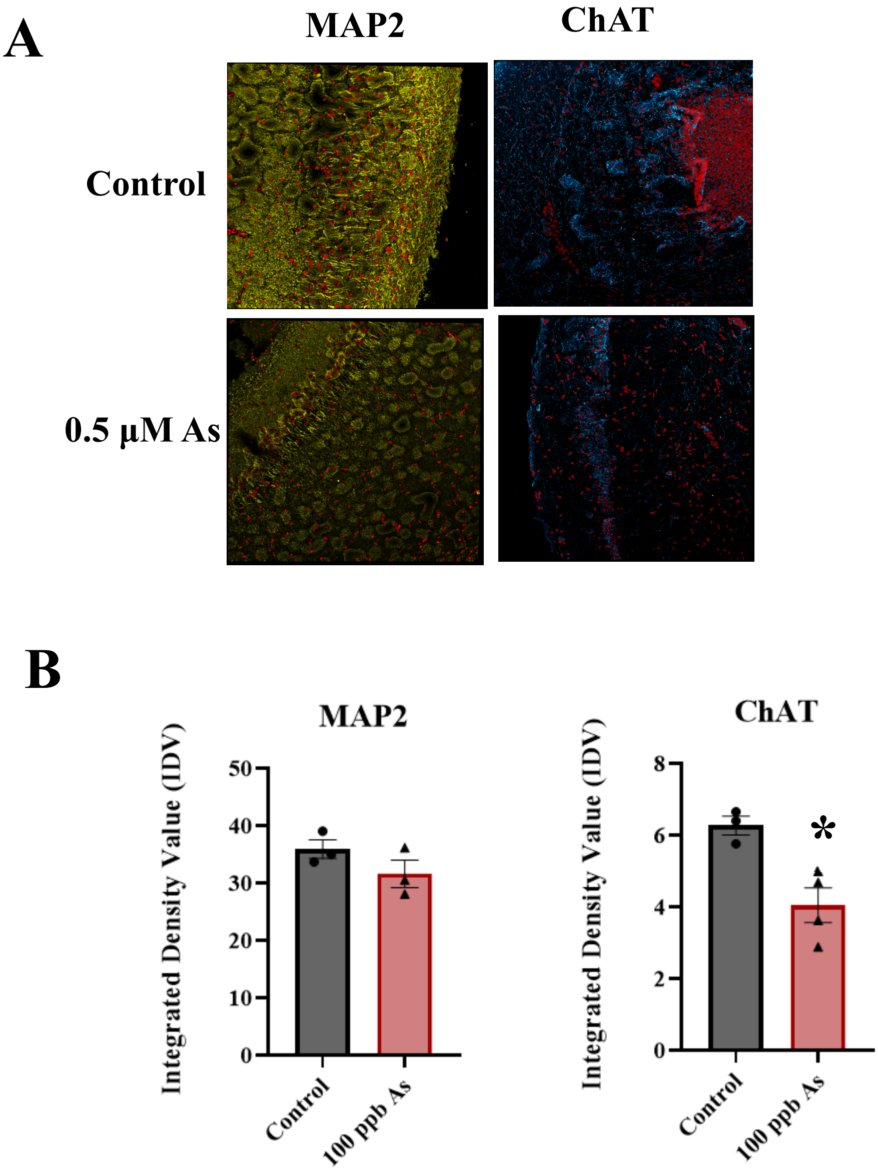

Supplement: Supplementary file 1 [file toxics-11-00644-s001.zip › toxics-2470922-supplementary.pdf]
